# Myelin down-regulates myelin phagocytosis by microglia and macrophages through interactions between CD47 on myelin and SIRPα (signal regulatory protein-α) on phagocytes

**DOI:** 10.1186/1742-2094-8-24

**Published:** 2011-03-15

**Authors:** Miri Gitik, Sigal Liraz-Zaltsman, Per-Arne Oldenborg, Fanny Reichert, Shlomo Rotshenker

**Affiliations:** 1 Dept. of Medical Neurobiology, IMRIC, Hebrew University Hadassah Medical School, P.O.B. 12272, Jerusalem 91120, Israel; 2 Dept. of Integrative Medical Biology, Umeå University, Umeå, Sweden

## Abstract

**Background:**

Traumatic injury to axons produces breakdown of axons and myelin at the site of the lesion and then further distal to this where Wallerian degeneration develops. The rapid removal of degenerated myelin by phagocytosis is advantageous for repair since molecules in myelin impede regeneration of severed axons. Thus, revealing mechanisms that regulate myelin phagocytosis by macrophages and microglia is important. We hypothesize that myelin regulates its own phagocytosis by simultaneous activation and down-regulation of microglial and macrophage responses. Activation follows myelin binding to receptors that mediate its phagocytosis (e.g. complement receptor-3), which has been previously studied. Down-regulation, which we test here, follows binding of myelin CD47 to the immune inhibitory receptor SIRPα (signal regulatory protein-α) on macrophages and microglia.

**Methods:**

CD47 and SIRPα expression was studied by confocal immunofluorescence microscopy, and myelin phagocytosis by ELISA.

**Results:**

We first document that myelin, oligodendrocytes and Schwann cells express CD47 without SIRPα and further confirm that microglia and macrophages express both CD47 and SIRPα. Thus, CD47 on myelin can bind to and subsequently activate SIRPα on phagocytes, a prerequisite for CD47/SIRPα-dependent down-regulation of CD47^+/+ ^myelin phagocytosis by itself. We then demonstrate that phagocytosis of CD47^+/+ ^myelin is augmented when binding between myelin CD47 and SIRPα on phagocytes is blocked by mAbs against CD47 and SIRPα, indicating that down-regulation of phagocytosis indeed depends on CD47-SIRPα binding. Further, phagocytosis in serum-free medium of CD47^+/+ ^myelin is augmented after knocking down SIRPα levels (SIRPα-KD) in phagocytes by lentiviral infection with SIRPα-shRNA, whereas phagocytosis of myelin that lacks CD47 (CD47^-/-^) is not. Thus, myelin CD47 produces SIRPα-dependent down-regulation of CD47^+/+ ^myelin phagocytosis in phagocytes. Unexpectedly, phagocytosis of CD47^-/- ^myelin by SIRPα-KD phagocytes, which is not altered from normal when tested in serum-free medium, is augmented when serum is present. Therefore, both myelin CD47 and serum may each promote SIRPα-dependent down-regulation of myelin phagocytosis irrespective of the other.

**Conclusions:**

Myelin down-regulates its own phagocytosis through CD47-SIRPα interactions. It may further be argued that CD47 functions normally as a marker of "self" that helps protect intact myelin and myelin-forming oligodendrocytes and Schwann cells from activated microglia and macrophages. However, the very same mechanism that impedes phagocytosis may turn disadvantageous when rapid clearance of degenerated myelin is helpful.

## Background

Oligodendrocytes in the central nervous system (CNS) and Schwann cells in the peripheral nervous system (PNS) form specialized myelin extensions of their plasma membranes that surround axons normally enabling them rapid conduction of electrical activity. Traumatic injury to axons produces abrupt breakdown of axons and myelin where physical trauma occurs. Then, axons and myelin also break down distal to the lesion as Wallerian degeneration (WD) develops [1]. Degenerated myelin is phagocytosed at injury sites in traumatized CNS by activated resident microglia and by activated blood-borne macrophages that gain access to the site through ruptured vasculature. In contrast, macrophages are not recruited and resident microglia are not activated to phagocytose degenerated myelin distal to lesion where CNS-WD develops [2-4]. Consequently, myelin-associated inhibitory molecules (e.g. Nogo, OMgp and MAG) impede axonal regeneration and repair; reviewed most recently in [5,6]. Blood-borne macrophages that are scarce in intact PNS are recruited and activated along with resident Schwann cells to remove degenerated myelin by phagocytosis during PNS-WD and regeneration of severed PNS axons follows [2,7,8]. However, PNS repair is often not successful in humans as it is in mice and rats [9,10]. This discrepancy has been attributed, in part, to the several-fold longer nerve segments that need to be cleared of degenerated myelin in humans. This results in delayed onset and slower axonal regeneration which contrasts with the prompt regeneration and reinnervation that are the most important determinants of good functional recovery. Therefore, revealing mechanisms that slow down myelin clearance is important.

**Figure 1:**
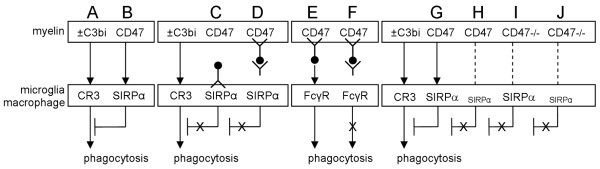
**Myelin regulates its own phagocytosis by simultaneous activation and down-regulation - a schematic representation of the working hypothesis and experimental design**. (A & B) Degenerated wild-type CD47^+/+ ^(CD47) myelin can simultaneously bind CR3 and SIRPα, which are expressed on microglia and macrophages. (A) Myelin binds CR3, which is the major receptor that mediates myelin phagocytosis in context of traumatic injury (see text), either directly or indirectly after being opsonized by complement protein C3bi (± C3bi). CR3 ligation initiates a signaling cascade that activates phagocytosis. (B) SIRPα ligation by myelin CD47 initiates a signaling cascade that inhibits phagocytosis by down-regulating signaling produced by CR3. (C & D) Augmentation of phagocytosis is anticipated if CD47 binding to SIRPα is blocked by either anti-SIRPα or anti-CD47 mAbs. (C) SIRPα on phagocytes is blocked by anti-SIRPα mAbs. (D) Myelin CD47 is blocked by anti-CD47 mAbs whose Fc-segments (black circle) are coated with anti-Fc-Fab_2 _fragments that lack their own Fc-segments. (E) Myelin CD47 is blocked by anti-CD47 mAbs whose Fc-segments are exposed. Consequently, CD47 binding to SIRPα is blocked (as in D) but binding to and activation of phagocytosis through FcγR is possible. (F) However, if anti-CD47 Fc-segments are coated by anti-Fc-Fab_2 _fragments (as in D), binding to and activation of phagocytosis through FcγR are blocked. Phagocytosis of (H) CD47^+/+ ^is expected to be augmented after reduction of SIRPα levels in phagocytes; i.e. compared to phagocytosis of CD47^+/+ ^by (G) phagocytes expressing normal SIRPα levels. Further, phagocytosis of CD47^-/- ^myelin by phagocytes expressing (I) normal or (J) reduced SIRPα levels are expected to be about the same.

We presently test the hypothesis that myelin regulates its own phagocytosis by simultaneous activation and down-regulation of microglia/macrophages (Figure 1A &1B). Activation follows myelin binding and subsequently activation of receptors that mediate its phagocytosis; CR3 (complement receptor-3), SRA (scavenger receptor-AI/II) and FcγR (Fcγ receptor) [11-15]. CR3 and SRA contribute most to phagocytosis in the absence of anti-myelin Abs, as is the case following axonal injury in-vivo and in our assay system. Further, of the two, CR3 contributes two- to three-fold more to phagocytosis than SRA. FcγR mediates myelin phagocytosis when anti-myelin Abs are present and opsonize myelin, as in multiple sclerosis. Down-regulation may follow myelin binding and subsequent activation of immune inhibitory receptors, which has not been previously studied. We specifically test here whether phagocytosis by macrophages and microglia is down-regulated after myelin-associated CD47 binds to the immune inhibitory receptor SIRPα on phagocytes.

SIRPα (CD172α, SHPS-1, p84, gp93, and BIT) is a member of an immune inhibitory family of receptors that down-regulate innate immune functions in myeloid cells; reviewed recently in [16-18]. SIRPα is expressed also on neurons. CD47, known also as IAP (integrin associated protein), is a SIRPα ligand and itself an SIRPα and thrombospondin receptor. CD47 is expressed on myeloid cells, RBCs (red blood cells), platelets, neurons, fibroblasts and endothelial cells [19,20]. CD47 expression on myelin and myelin-forming oligodendrocytes and Schwann cells has not been previously reported.

CD47-SIRPα binding requires cell-cell contact since both are cell membrane protein receptors. Previous reports have documented that RBCs and platelets that were opsonized by Ab/IgG or complement protein C3bi down-regulate their own phagocytosis by FcγR and CR3 in macrophages after CD47 on RBCs and platelets binds SIRPα on phagocytes. These observations led to the classification of CD47 as a marker of "self" whose function is to protect intact cells from being phagocytosed by autologous macrophages [21-24].

We presently demonstrate that wild-type myelin that expresses CD47 (CD47^+/+^) down-regulates its own phagocytosis by microglia and macrophages after myelin CD47 binds SIRPα on phagocytes. CD47 may function, therefore, as a marker of "self" in myelin and myelin-forming oligodendrocytes and Schwann cells. We further document that components in serum may also promote SIRPα-dependent down-regulation of myelin phagocytosis irrespective of myelin CD47.

## Methods

**Animals **Sprauge-Dawley rats and wild-type and CD47^-/- ^Balb/C mice that were obtained from Harlan (Israel) and [21] were handled in accordance with the national research council's guide for the care and use of laboratory animals and the approval of the institutional committee.

**Figure 2:**
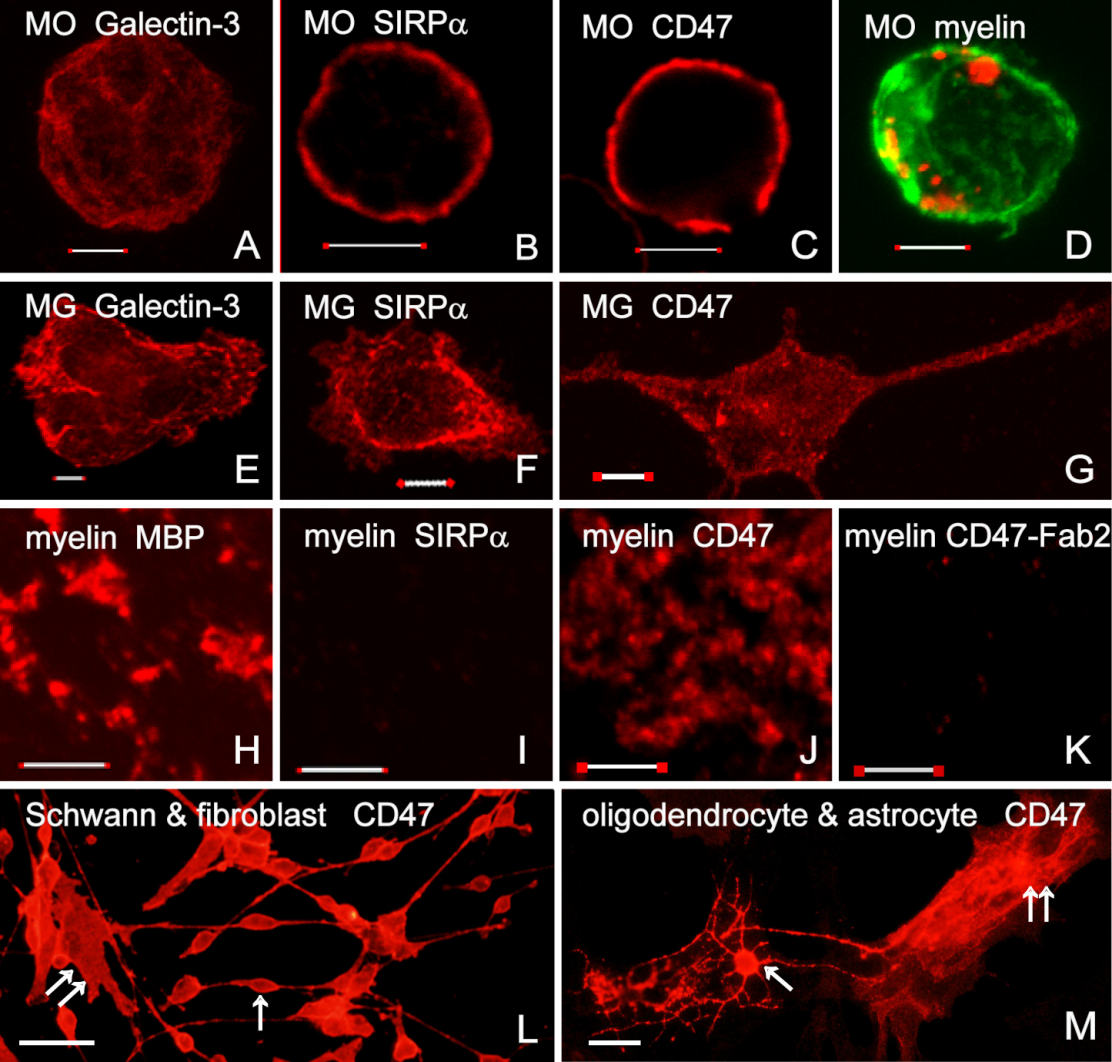
**Macrophages and microglia express CD47 and SIRPα whereas myelin, Schwann cells, oligodendrocytes and astrocytes express CD47 without SIRPα.** Macrophages (MO) express (A) Galectin-3, cell surface (B) SIRPα, and (C) CD47. (D) Macrophages phagocytose myelin. F-actin (filamentous actin) is visualized by Alexa 488 labeled phalloidin (green) and myelin by anti-MBP mAb (red); overlap between the two is yellow. Myelin is present in the cytoplasm interior to cortical F-actin. Similar observations were made in microglia (not shown; see [29]). Microglia (MG) express (E) Galectin-3, and cell surface (F) SIRPα and (G) CD47. (H) MBP and (J) CD47 are expressed in myelin without (I) SIRPα. (K) Anti-Fc-Fab_2 _fragments coat/block Fc-segments of anti-CD47 mAb that binds CD47 on myelin, thus preventing visualization of anti-CD47 mAb by a secondary Ab. (L) CD47 is expressed on spindle shaped bipolar Schwann cells (arrow) and flat fibroblasts (double arrow). (M) CD47 is expressed on oligodendrocytes (arrow) that extend elongated branched processes, and on flat astrocytes (double arrow). Galectin-3, CD47, SIRPα and MBP are visualized by immunofluorescence confocal microscopy using respective mAbs directed against each (red). Bars are 5 μm in A through K, and 50 μm in L and M.

**Thioglycollate-elicited peritoneal primary macrophages **were harvested in cold DMEM/F12 3 to 4 days after intraperitoneal injection of 75μL/gram body weight of 3% thioglycollate (Difco, USA), and plated in the presence of 10% heat-inactivated FCS in 96-well culture plates (Nunc International, USA) for 2 hours. Non-adherent cells were washed away. The remaining adhered cells are macrophages that express Galectin-3 (formally known as MAC-2) (Figure 2A), CR3 and F4/80 [8,25,26].

**Primary microglia **were isolated from brains of neonatal mice and rats as previously described [11]. In brief, the brains were stripped of their meninges and enzymatically dissociated, and cells were then plated on poly-L-lysine coated flasks for one week and replated for 1 to 2 hours on bacteriological plates. Non-adherent cells were then washed away. The vast majority of adherent cells are microglia judged by morphology and positive immunoreactivity to Galectin-3/MAC-2 (Figure 2E), CR3 and F4/80 [4,27].

**Mixed cell cultures; Schwann cells and fibroblasts from PNS and astrocytes and oligodendrocytes from CNS **were obtained as previously described [4,8,28]. In brief, neonate brains (i.e. CNS) and sciatic nerves (i.e. PNS) were cut into small pieces. The brains were incubated for 1 hour in DMEM containing 0.25% trypsin. The nerves were incubated for 18 to 20 hours in DMEM containing 1.2 units/ml dispase (Boehringer), 0.05% collagenase and 0.1% hyaluronidase (Sigma, Israel). Dissociated tissues were plated on laminin-coated culture dishes or bacteriological plates. Cells were identified by their source, morphology and immunoreactivity to specific markers. In PNS cultures [8,28], Schwann cells displayed bipolar, spindle-shaped morphology, were S-100- and Galectin-3/MAC-2-positive but F4/80- and CR3-negative. Fibroblasts were flat, F4/80-, CR3-, S-100- and Galectin-3/MAC-2-negative cells. In CNS cultures [4], astrocytes were GFAP-positive but F4/80-, CR3-, Galectin-3/MAC-2- and Gal-C-negative. Oligodendrocytes displayed a characteristic arbor of thin, elongated, branched processes and were Gal-C-positive but GFAP-, F4/80-, CR3- and Galectin-3/MAC-2-negative.

### Myelin phagocytosis

Microglia and macrophages were plated in 96-well tissue culture plates at a density that minimizes cell-cell contact (0.25-1.5 × 10^4^/well) in the presence of DMEM supplemented by 10% HI-FCS (heat inactivated FCS). Non-adherent cells were washed out after 2 hours and adherent phagocytes left to rest overnight. Then phagocytes were washed, myelin added for 60 minutes in the presence of serum in DMEM/F12, unphagocytosed myelin washed out, and levels of phagocytosis determined by ELISA (see below). At this point remaining myelin has already been phagocytosed/internalized (Figure 2D; see also [29]). When testing phagocytosis in the presence of anti-SIRPα mAb (2 to 5 μg/ml), phagocytes were pre-incubated in the presence of mAb or matched control IgG in triplicates for 30 minutes and phagocytosis assayed in their continuous presence at 37°C (Figure 1C). When testing phagocytosis of myelin whose CD47 was blocked by anti-CD47 mAb, myelin was first pre-incubated with anti-CD47 (5 μg/ml) or matched control IgG in a ratio of 1:1 for 30 minutes at 37°C and unbound mAb washed away. However, before blocking CD47 on myelin, Fc-segments of anti-CD47 and control IgG were blocked/coated by incubating them with Fab_2 _fragments of goat anti-mouse IgG in a ratio of 1:5 for 30 minutes at 37°C. Myelin opsonized by Fc-coated anti-CD47 or Fc-coated control IgG was added to phagocytes and phagocytosis assayed (Figure 1D). When testing phagocytosis in the absence of serum, phagocytes were washed and incubated in serum-free media supplemented by 0.1% delipidated BSA (MP Biomedicals, Inc) for 4 hours, washed, and phagocytosis was then assayed in serum-free DMEM/F12 supplemented by 0.1% delipidated BSA.

### ELISA assay to quantify myelin phagocytosis

This assay is based on the detection of myelin basic protein (MBP) in macrophages and microglia lysates. Since MBP is unique to PNS and CNS myelin and is not produced by phagocytes, MBP levels detected in phagocyte cytoplasm are proportional to levels of myelin phagocytosed. In brief, after non-phagocytosed myelin was washed away and remaining myelin had already been phagocytosed (Figure 2D), phagocytes were lysed (0.05 M carbonate buffer, pH 10), the lysates transferred to high protein absorbance plates (Nalge Nunc International, USA), and levels of MBP were determined by ELISA using anti-MBP mAbs. A detailed protocol is given in [26] where we have also determined that more than 95% of the detected MBP arises from phagocytosed myelin (see also Figure 2D). We further verified the validity of this phagocytosis assay by testing the ability to inhibit myelin phagocytosis down to 5% of control by cytochalasin-D (not shown).

Quantification of phagocytosis was carried out in the following way. When phagocytosis by microglia infected with SIRPα-shRNA (SIRPα-knocked-down; SIRPα-KD microglia) was compared to phagocytosis by control microglia infected with non-target Luciferase-shRNA (Con-Luc microglia), phagocytosis by each was first normalized to the number of respective microglia counted in 1-mm^2 ^areas at the center of wells. Normalizing phagocytosis to cell number is required since the number of adherent microglia could differ because SIRPα-KD and Con-Luc microglia may differ in adhesion properties to plates. To this end, microglia in replicate plates were fixed (i.e. instead of being lysed for the phagocytosis assay), stained and counted. Then, phagocytosis, normalized to cell number by SIRPα-KD microglia, was calculated as a percentage of phagocytosis normalized to cell number by Con-Luc microglia, which was defined as 100%. When phagocytosis by wild-type microglia was tested in the presence of anti-SIRPα, anti-CD47 or control IgG (see above), phagocytosis in the presence of either anti-SIRPα or anti-CD47 was calculated as percentage of phagocytosis in control IgG, which was defined as 100%. In this case, phagocytosis was not normalized to cell number since we confirmed that the number of adherent cells did not differ between wells, as the same population of wild-type microglia was tested in all wells.

**Myelin isolation **from mouse and rat brains has been previously described [13,26].

### Media products

DMEM, DMEM/F12, FCS, Gentamycin sulfate, and L-Glutamine were obtained from Biological Industries (Beit-Haemek, Israel).

### Immunocytochemistry

Cells or myelin were plated, fixed for 20 min at RT in 4% Formaldehyde in PBS, washed, blocked over night at 4°C in 10% FCS in PBS, incubated for 1.5 hours at 37°C in either anti-CD47 (5 μg/ml), anti-SIRPα (5 μg/ml), or anti-MBP (2 μg/ml), washed, incubated in Cy3 labeled rabbit anti-mouse Abs for 40 minutes at RT, washed and mounted. For combined myelin and F-actin staining, fixed cells were permeabilized for 10 minutes at RT in 0.01% Triton × -100 (Sigma Aldrich, Israel) in PBS, washed, blocked for 2 hours at 37°C in 10% FCS in PBS, incubated for 1.5 hours at 37°C in anti-MBP (2 μg/ml), washed, incubated in Cy3 labeled donkey anti-rat Abs and alexa fluor 488 phalloidin (1:100) for 40 minutes, washed and mounted. For Galectin-3 staining, permeabilized and blocked cells were incubated for 1.5 hours at 37°C in mouse anti-rat Galectin-3 (4 μg/ml), washed and incubated for 40 minutes at RT in Cy3-labeled rabbit anti-mouse, washed and mounted.

### Generation of microglia with stable reduced SIRPα expression

Reduction of SIRPα expression was achieved through lentiviral infection of wild-type Balb/C microglia with short hairpin RNAs directed against mouse SIRPα mRNA (SIRPα-shRNA) using pLKO.1 puro plasmids (Sigma, Israel). Four different shRNA sequences were used. All were effective in reducing SIRPα expression and the one selected for this study is in the SIRPα cDNA coding sequence 5'CCGGTGGTTCAAAGAACTCGAGTTCTTGCCCATCTTTGAACCATTTTTG-3'. The plasmid was transfected into a 293T-based packaging cell line, and the resulting culture supernatant was used for lentiviral infection. Infected microglia were selected on the basis of their resistance to puromycin brought by the pLKO.1 plasmid. The level of SIRPα protein expression was monitored by western blotting. As a control, microglia were infected in a similar way with the shRNA sequence 5'CTTACGCTGAGTACTTCGA-3' against the non-target firefly Luciferase gene (a gift from Dr I. Ben-Porath).

### Antibody source

Mouse anti-rat CD47 and SIRPα/CD172α mAbs, rat anti-mouse MBP mAb and matched control IgG - Serotec (Oxford, England), anti-rat Galectin-3 mAb - Santa Cruz (USA), rat anti-mouse Galectin-3 mAb (M3/38 hybridoma cell line; American Type Culture Collection, Rockville, MD, USA), Cy3-labeled rabbit anti-mouse, Fab_2 _fragment goat anti-mouse IgG, and anti-normal rat IgG - Jackson (West Grove, USA).

**Confocal fluorescence microscopy **was carried out using an Olympus FluoView FV1000 confocal microscope. Alexa Fluor 488-labeled phalloidin (Molecular Probes, USA) was used to visualize F-actin. Optical slices of phagocytes, 1 μm thick, were scanned sequentially.

### Statistical analysis

Non-parametric Mann-Whitney analysis was carried out and all *p*-values of significance are two tailed. Values of individual experiments as well as averages ± SE are given.

## Results

### CD47 and SIRPα are expressed on macrophages and microglia, and CD47 without SIRPα on myelin, Schwann cells and oligodendrocytes

A pre-requisite for wild-type myelin inhibiting its own phagocytosis through CD47-SIRPα interactions (Figure 1A &1B) is the expression of CD47 on myelin and SIRPα on macrophages and microglia. We examined, therefore, whether CD47 and/or SIRPα are expressed on myelin and myelin-forming Schwann cells and oligodendrocytes, which has not been previously reported. We further sought to confirm that phagocytes used in this study express CD47 and SIRPα. Rat and mouse microglia, astrocytes, oligodendrocytes and myelin were isolated from brains, Schwann cells and fibroblasts from sciatic nerves, and thioglycollate-elicited macrophages from the peritoneal cavity. The identity of each cell type was verified by its source, morphology and specific markers (see Methods). Amongst these we present here are positive immunoreactivity to Galectin-3/MAC-2 in macrophages and microglia (Figure 2A &2E) and to MBP in myelin (Figure 2H). Macrophages and microglia are also immunoreactive to both SIRPα and CD47 (Figure 2B, C, F &2G). Myelin, Schwann cells, oligodendrocytes, astrocytes and fibroblasts are immunoreactive to CD47 but not SIRPα (Figure 2I, J, L &2M). Negative immunoreactivity to SIRPα in Schwann cells, oligodendrocytes, fibroblasts and astrocytes is not shown. Similar patterns of Galectin-3/MAC-2, CD47 and SIRPα expression were detected in mouse and rat cells. We chose to present data from rat macrophages, microglia and myelin (Figure 2A through 2K) since those were used in the forthcoming experiment in which we test how blocking binding between CD47 and SIRPα influences phagocytosis.

### Myelin phagocytosis is down-regulated after myelin CD47 binds to SIRPα on macrophages and microglia

Down-regulation of myelin phagocytosis, which depends on binding between CD47 on myelin and SIRPα on phagocytes, suggests augmentation of phagocytosis when CD47-SIRPα binding is blocked by mAbs directed against either one (Figure 1C &1D). We used rat microglia and macrophages to study phagocytosis in the presence of function-blocking mouse anti-rat CD47 and SIRPα mAbs since those are commercially available. We could not use mouse phagocytes in this experiment since rat anti-mouse CD47 and SIRPα mAbs that are useful for immunocytochemistry do not block function.

**Figure 3:**
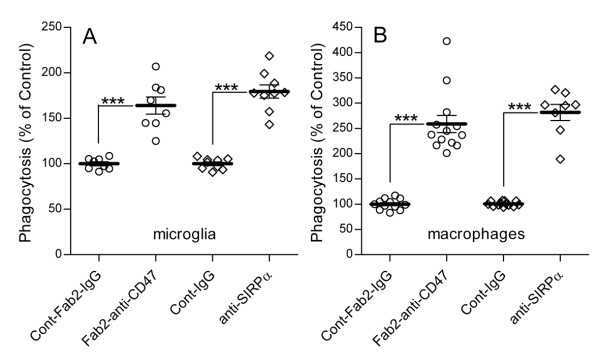
**Myelin phagocytosis is augmented in the presence of anti-CD47and anti-SIRPα function-blocking mAbs (see also Fig. 1)**. Myelin phagocytosis is augmented in (A) microglia and (B) macrophages in the presence of function-blocking anti-SIRPα and anti-CD47 mAbs. Phagocytosis was assayed using different paradigms for each mAb (see text). (i) Microglia and macrophages were pre-incubated in the presence of anti-SIRPα or matched control IgG (Cont-IgG), myelin was added and phagocytosis was assayed. Phagocytosis in the presence of anti-SIRPα was calculated as a percentage of the phagocytosis in control IgG, which was defined as 100%. (ii) Anti-CD47 or matched control IgGs that had their Fc-segments coated/blocked by anti-Fc Fab_2 _fragments (Fab2-anti-CD47 and Cont-Fab2-IgG, respectively) were used to opsonize myelin that was added to phagocytes. Phagocytosis of myelin opsonized by Fab_2_-anti-CD47 was calculated as a percentage of phagocytosis in the presence of Cont-Fab_2_-IgG, which was defined as 100%. Values of individual experiments, each performed in triplicates, as well as averages ± SE are given; two tailed p-values of significance are *** p < 0.001.

We blocked first SIRPα on rat microglia and macrophages by pre-incubating phagocytes with mouse anti-rat SIRPα mAb or matched control IgG for 30 minutes (Figure 1C). Myelin was then added and phagocytosis tested in the continuous presence of anti SIRPα or control IgG. Phagocytosis by macrophages and microglia was augmented 280% and 180% of control, respectively (Figure 3).

We then blocked CD47 on myelin before providing it to microglia and macrophages. Myelin was pre-incubated in the presence of mouse anti-rat CD47 mAb or matched control IgG for 30 minutes and unbound mAb/IgG washed out. This procedure may enable FcγR-mediated phagocytosis of myelin that is opsonized by mAb/IgG (Figure 1E). We blocked, therefore, Fc-segments of anti-CD47 and control IgG to exclude phagocytosis by FcγR (Figure 1F). To this end, mouse anti-rat CD47 mAb and matched control IgG were incubated with Fab_2 _fragments of goat anti-mouse IgG that lack their own Fc-segments. To verify coating/blocking efficiency, myelin was opsonized by either Fc-coated or uncoated anti CD47. Bound anti-CD47 was visualized by immunofluorescence confocal microscopy using Cy3-labelled anti-mouse Ab/IgG, which can bind Fc-uncoated mouse anti-rat CD47 (Figure 1E) but not Fc-coated mouse anti-rat CD47 (Figure 1D &1F). Myelin opsonized by Fc-uncoated anti-CD47 displayed positive immunoreactivity (Figure 2J) whereas myelin opsonized by Fc-coated anti CD47 did not (Figure 2K). Thus anti-Fc-Fab_2 _coated/blocked most, if not all, Fc-segments of anti CD47.

Myelin opsonized by Fc-coated anti-CD47 or Fc-coated matched control IgG was added to macrophages and microglia and phagocytosis assayed. Phagocytosis of myelin opsonized by Fc-coated anti CD47 was augmented in both macrophages and microglia 260% and 160% of control, respectively (Figure 3). Thus, levels of augmentation in the presence of anti-CD47 and anti-SIRPα were similar.

### Myelin CD47 and serum can each promote SIRPα-dependent down-regulation of myelin phagocytosis in microglia

**Figure 4:**
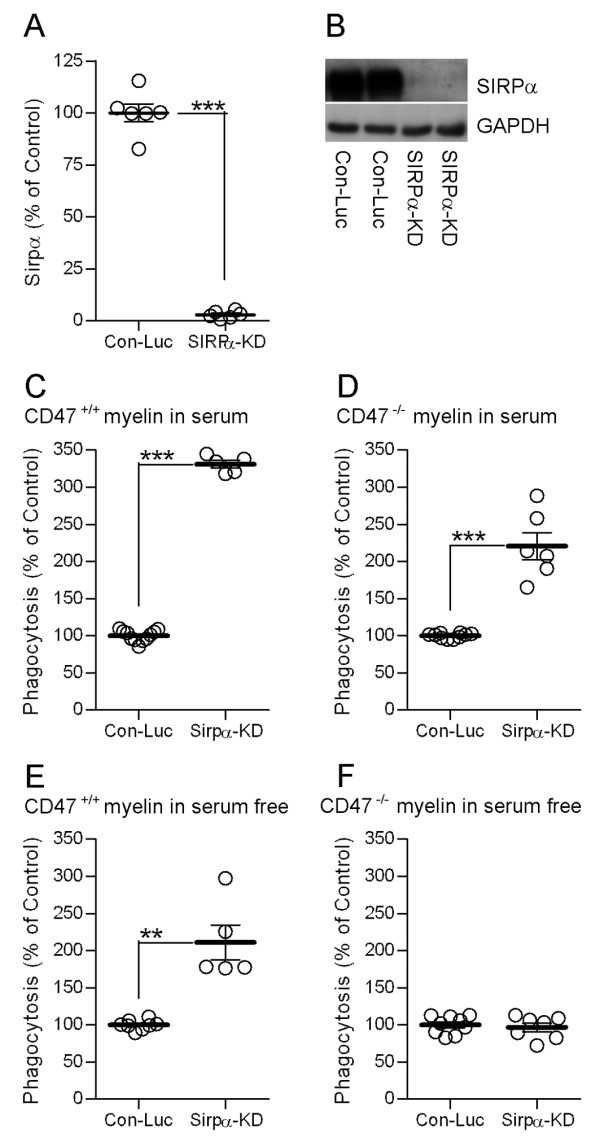
**CD47 on myelin and serum can each promote SIRPα-dependent down-regulation of myelin phagocytosis in microglia with reduced SIRPα levels.** (A) Quantitation of SIRPα levels in Balb/C microglia infected with SIRPα-shRNA (SIRPα-KD) or non-target control Luciferase-shRNA (Con-Luc) based on (B) immunoblot analyses in which SIRPα and GAPDH levels were determined. An SIRPα to GAPDH ratio in SIRPα-KD microglia was calculated as a percentage of that ratio in Con-Luc microglia, which was defined as 100%. Values of individual experiments, averages ± SE and levels of significance are given; two tailed p-value of significance is *** p < 0.001. Phagocytosis of CD47^+/+ ^myelin is augmented in SIRPα-KD microglia compared to phagocytosis by Con-Luc microglia in both (C) the presence and (E) the absence of serum. Phagocytosis of CD47^-/- ^myelin is augmented in SIRPα-KD microglia compared to phagocytosis by Con-Luc microglia in (D) the presence but not (F) the absence of serum. Phagocytosis by SIRPα-KD microglia was calculated as a percentage of phagocytosis by Con-Luc microglia, which was defined as 100% (see Methods). Values of individual experiments, each performed in triplicates, averages ± SE and levels of significance are given; two tailed p-value of significance are ** p < 0.01 and *** p < 0.001.

SIRPα-dependent down-regulation of CD47^+/+ ^myelin phagocytosis suggests augmentation of phagocytosis after reducing SIRPα levels in phagocytes (Figure 1G &1H). We knocked-down SIRPα levels (SIRPα-KD) in wild-type primary Balb/C microglia by Lentiviral infection with SIRPα-shRNA, down to 3% of levels in control microglia that were infected with non-target Luciferase-shRNA (Con-Luc; Figure 4A &4B). Concurrently, phagocytosis of CD47^+/+ ^myelin was augmented in SIRPα-KD microglia 330% of control (Figure 4C). It is further expected that this augmentation will not take place if myelin does not express CD47 (Figure 1I &1J). However, contrary to prediction, phagocytosis of CD47^-/- ^myelin was augmented in SIRPα-KD microglia 220% of control (Figure 4D), suggesting augmentation that is dependent on SIRPα but independent of myelin CD47. Since experiments were carried out thus far in medium supplemented by serum, it is possible that components in serum may have activated SIRPα directly or indirectly through transactivation [30]. To exclude serum dependent activation of SIRPα, experiments were repeated in serum-free medium supplemented by 0.1% delipidated BSA. Phagocytosis of CD47^+/+ ^myelin was augmented in SIRPα-KD microglia 210% of control (Figure 4E), indicating that CD47 on myelin can promote SIRPα-dependent down-regulation of myelin phagocytosis irrespective of serum. Further, phagocytosis of CD47^-/- ^myelin in the absence of serum was not altered from control (Figure 4F), indicating that augmentation of phagocytosis of CD47^-/- ^myelin in the presence of serum (Figure 4D) was serum-dependent. Thus serum can promote SIRPα-dependent down-regulation of myelin phagocytosis irrespective of CD47 on myelin.

## Discussion

This study provides evidence that myelin regulates its own phagocytosis by simultaneous activation and down-regulation in macrophages and microglia (Figure 1A &1B). Activation follows myelin binding/activating receptors that mediate its phagocytosis (e.g. CR3 and SRA; [11-15]). Down-regulation follows myelin CD47 binding to and activation of SIRPα on macrophages and microglia, which we document here for the first time. Further, myelin CD47 and serum can each promote SIRPα-dependent down-regulation of myelin phagocytosis irrespective of the other.

Down-regulation of myelin phagocytosis that depends on myelin CD47 binding to and activation of SIRPα on phagocytes is suggested by the following observations: CD47 is expressed normally on myelin and SIRPα is expressed normally on microglia and macrophages (Figure 2), which is a pre-requisite for SIRPα activation in phagocytes by myelin CD47; phagocytosis of CD47^+/+ ^myelin is augmented after blocking binding between CD47 and SIRPα (Figure 3), indicating that down-regulation of phagocytosis indeed follows CD47-SIRPα binding; phagocytosis of CD47^+/+ ^myelin is augmented after reducing SIRPα levels in phagocytes (Figure 4C &4E), suggesting that down-regulation of phagocytosis depends on SIRPα in phagocytes; and, finally, phagocytosis in serum-free medium of CD47^-/- ^myelin is not altered from normal in phagocytes with reduced SIRPα levels (Figure 4F), suggesting that myelin CD47 produces SIRPα dependent down-regulation of CD47^+/+ ^myelin phagocytosis irrespective of serum (Figure 4E &4F).

Further, serum can promote SIRPα-dependent down-regulation of myelin phagocytosis irrespective of myelin CD47, although both serum and CD47 can produce this down-regulation at the same time. This is suggested by the following observations: phagocytosis of CD47^-/- ^myelin by phagocytes with reduced SIRPα levels is augmented in the presence of serum but not in its absence (Figure 4D &4F), indicating that removal of serum alleviates SIRPα-dependent down-regulation of phagocytosis in the absence of myelin CD47; further, augmentation of phagocytosis of CD47^+/+ ^myelin by phagocytes with reduced SIRPα levels in the presence of serum exceeds phagocytosis augmentation in the absence of serum (Figure 4C &4E), suggesting that reducing SIRPα levels alleviate both CD47- and serum-induced, SIRPα-dependent down-regulation of phagocytosis. We did not examine in this study how serum promotes SIRPα-dependent down-regulation of myelin phagocytosis. Potential mechanisms could be transactivation of SIRPα by other receptor(s) or by as yet unidentified soluble SIRPα ligand(s) in serum. For example, SIRPα may be transactivated by EGFR whose soluble ligands are present in serum, as has been reported in other systems [30]. Further studies are required to elucidate those mechanisms that underlie serum/SIRPα-dependent down-regulation of phagocytosis.

CD47/SIRPα-dependent inhibition of FcγR- and CR3-mediated phagocytosis of IgG- and C3bi-opsonized RBCs and platelets has been previously documented in macrophages [21-24]. These observations have led to the notion that CD47 functions as a marker of "self," whose physiological role is to protect intact CD47^+/+ ^RBCs and platelets from activated autologous macrophages that express SIRPα. Our present observations suggest that CD47 may function also as marker of "self" on myelin and myelin-forming Schwann cells and oligodendrocytes, as CD47 may help protect these cells from activated macrophages and microglia. This mechanism could be helpful under normal conditions and in infectious diseases where phagocytes need to be activated to phagocytose pathogens but bystander intact "self" cell populations should be spared.

However, this very same mechanism may turn disadvantageous after traumatic injury to PNS and CNS axons and in multiple sclerosis. Rapid phagocytosis of degenerated myelin, which CD47-SIRPα interaction impedes, is useful for repair after traumatic injury to PNS axons, especially in humans; see Background and [9,10]. The same argument holds for repair after CNS axonal injury in conjunction with attempts to override myelin-dependent inhibition of regeneration [31-34]. In multiple sclerosis, degenerated myelin activates the complement system to form membrane attack complexes, which then produce damage to intact axons and myelin and further inhibit remyelination [35-38]. Rapid removal of degenerated myelin, which CD47-SIRPα interaction impedes, may harness these detrimental effects.

## Conclusions

Myelin phagocytosis is regulated by activation and inhibitory mechanisms, suggesting that the balance between these two determines how rapid and efficient phagocytosis will be. Inhibitory mechanisms, such as the CD47-SIRPα interaction that we document here, are useful under normal conditions and while combating invading pathogens to protect intact myelin and myelin-forming oligodendrocytes and Schwann cells from activated microglia and macrophages. The very same mechanisms may turn harmful when faster removal of degenerating myelin is useful (e.g. after traumatic injury and in multiple sclerosis). Further, SIRPα-dependent down-regulation of phagocytosis may also be promoted by serum.

## Abbreviations

Con-Luc: control Luciferase; CR3: complement receptor-3; FcγR: Fcγ receptor; KD: knock-down; mAb: monoclonal antibody; MBP: myelin basic protein; shRNA: short hairpin RNA, SIRPα: signal regulatory protein-α; SRA: scavenger receptor-AI/II; WD: Wallerian degeneration.

## Competing interests

The authors declare that they have no competing interests.

## Authors' contributions

MG carried out all experiments, SLZ carried out some of the immunocytochemistry and function blocking experiments, PAO provided rat anti-mouse CD47 and SIRPα mAbs and CD47^-/- ^mice, FR participated in most experiments, SR is the group leader responsible for the design and analysis of experiments and manuscript preparation. All authors read and approved the final manuscript.
